# Advancing pediatric anesthesia in Brazil: reflections on research and education

**DOI:** 10.1016/j.bjane.2024.844535

**Published:** 2024-06-25

**Authors:** Norma Sueli Pinheiro Módolo, Débora de Oliveira Cumino, Luciana Cavalcanti Lima, Guilherme Antonio Moreira de Barros

**Affiliations:** a Universidade Estadual Paulista (UNESP), Faculdade de Medicina de Botucatu (FMB), Departamento de Especialidades Cirúrgicas e Anestesiologia, Botucatu, SP, Brazil; b Sabará Hospital Infantil, São Paulo, SP, Brazil; c Serviço de Anestesiologia Pediátrica/SAPE, Brazil; d Hospital Municipal Menino Jesus, São Paulo, SP, Brazil; e Instituto Medicina Integral Professor Fernando Figueira, Recife, PE, Brazil; f Faculdade Pernambucana de Saúde, Recife, PE, Brazil

Conducting research is a complex task. In pediatrics, in particular, there are obstacles that further complicate its execution, considering that the pediatric population presents particularities in physiology and development that, consequently, are reflected in pharmacodynamics and pharmacokinetics.^1^, ^2^, ^3^, ^4^ In addition to the fact that a child is not a miniature adult, there are other difficulties that include and surpass regulatory issues and impose restrictions on studies.^1^

There are technical aspects that hinder the recruitment of children, such as the small number of individuals with serious medical problems that justify the conduction of specific studies, the need for appropriate outcome evaluation for different ages, and the required adaptations in procedures and research environments to accommodate different levels of physical, cognitive, and emotional development. There are also few researchers specialized in various areas of pediatrics, with the knowledge and qualifications necessary to carry out age-appropriate procedures for participants. It is worth noting that there is limited infrastructure in research centers, including the limited availability of materials, equipment, and techniques, such as the use of small volumes in sample tests and the availability of validated monitors for children.^5^

Research in children also involves highly relevant ethical issues, which can be grouped into two dimensions: morality and legality.^6^ Ethical regulations in research were created to guide researchers' conduct and protect research subjects. These regulations were implemented in response to research involving children with intentional exposure to risks, without any benefit or clarification of potential unfavorable outcomes to their guardians. Thus, the primary objective of regulations is the protection of research participants, especially those belonging to vulnerable populations, such as children.^4^

The first international regulation created was the Nuremberg Code in 1947, in response to the research conducted in concentration camps. This was followed by the Declaration of Helsinki in 1964, which made it possible for vulnerable individuals to participate, provided their legal representative gave official consent. In 1993, the International Ethical Guidelines for Biomedical Research Involving Human Subjects were published, mandating the attainment of informed consent from all research participants and regulating risk-benefit evaluation, data confidentiality, and ethical and scientific review by committees.^6^^,^^7^

In Brazil, Resolution 196/1996 of the National Health Council regulated research involving human subjects and referred to children as a vulnerable population. This was repealed and replaced by Resolution 466/2012, which emphasizes the need for research to adhere to the principles of autonomy, beneficence, nonmaleficence, justice, and equity. Thus, the risks to which children may be subjected to as research subjects must be well defined and clearly communicated to their legal guardians and to the children themselves if they have sufficient cognitive development. The Research Ethics Committee must rigorously evaluate protocols involving children, including the requirement of obtaining the Assent Term from the participating child.^6^^,^^8^

On the other hand, children are considered therapeutic orphans due to the lack of drugs developed for this age group and the absence of age-appropriate formulations and administration routes that allow for accurate, safe, and palatable use. Specific research in pediatrics is needed because it is difficult to predict the ideal dose, route of administration, absorption, metabolism, elimination, and toxicity, among other characteristics of a drug, before conducting the first clinical trial.^1^, ^2^, ^3^, ^4^, ^5^ However, there has always been a preference for recruiting adult participants in research.^2^^,^^9^

For these reasons, many techniques and drugs used in daily pediatric practice are off-label.^10^ There is little financial interest from the pharmaceutical industry in developing medications for the pediatric age group because, unlike adults, children are mostly healthy, with diseases that can be controlled by inexpensive medications. Additionally, they require greater monitoring and more individuals involved in research, implying higher costs, and if the result is unfavorable, the repercussions will be negative and there is a risk of legal proceedings. If there is a need to repair harm, this will take much longer than in research recruiting adults.^3^^,^^5^

At the time of recruitment, legal representatives need to be convinced that the benefits outweigh the risks, and many of them feel uncomfortable allowing participation in the research. For children who have a greater understanding, the Assent Form must also be obtained and explained in simple and easily understandable language.^8^ Other factors that negatively impact recruitment include the fear of needles, blood collection, the taste of the medication, and the disruption of normal activities, such as attending school.^3^

We need to discipline ourselves to see things from various perspectives. It is necessary to understand all sides of the issue when admitting, or not, the use of off-label drugs in pediatric anesthesia, which depends on the likely advantages or risks of their use. From the child's perspective, off-label drugs can represent a more current and effective therapeutic option. From the parents' perspective, they may represent untested and unknown risks, which can concern and make them hesitant. From the federal authority's perspective, they represent serious concerns based, in part, on unknown risks and complications from the use of drugs that the regulatory agency does not control. From the industry's perspective, off-label drugs substantially increase annual sales without investment in research. From the anesthesiologist's perspective, these drugs can establish a standard of care with minimal risks or even improve it with greater safety, based on the best available scientific evidence.^11^

Educational efforts, supporting research, and promoting collaboration among physicians, patients’ families, and professionals are essential. In this regard, we currently observe remarkable national and international multi-institutional collaborations that allow for information exchange, large amounts of data collection, guideline development, and practice changes that drive the specialty forward. These collaborations are important for continuous improvement in the care of children.^4^ However, innovations to improve clinical care, greater investments in pediatric anesthesia research, and training and education of professionals specializing in child care are still required.^12^

In this context, specialization in pediatric anesthesia is dedicated to improving patient safety in this vulnerable population. Every day, pediatric anesthesiologists care for fragile children and their families during difficult times, striving to promote safety and quality in anesthetic procedures, perioperative care, and pain management. Various improvements have contributed to reduced anesthesia-related mortality rates over the past 50 years. However, children are known to be at greater risk for anesthetic complications during the perioperative period.^13^ In particular, anesthesia in neonates and infants poses challenges even for experienced anesthesiologists, especially for premature infants or those with comorbidities.^14^

The rates of cardiac arrest attributable to anesthesia in children are inversely related to the Human Development Index (HDI) of the country. However, regardless of the HDI, the rate of anesthesia-related cardiac arrest is higher in children under one year of age, indicating that safety has not improved for this population. On the other hand, the 24-hour survival rate after cardiac arrest increases as the HDI of the country increases. This progress can be attributed to advances in pharmacology, improvements in education and training in pediatric anesthesiology, increased availability of neonatal and pediatric intensive care services, centralization of care in specialized pediatric services, and investment in research directed at children. These facts highlight the need to fill this growing gap in anesthetic care for children.^15^

In developed countries, pediatric anesthesia is often a subspecialty of anesthesiology. Conversely, in underdeveloped and developing countries, the subspecialty scarcely exists, and anesthesiologists with little experience in pediatric anesthesia are often responsible for the care provided to children.^15^

In Brazil, significant differences in pediatric anesthesia training are observed, primarily due to the lack of recognition of the subspecialty, as well as the poor distribution or even nonexistence of specialized services in various regions of this extensive country.^16^ During anesthesiology training, pediatric anesthesia training is limited and consequently inadequate in terms of knowledge, competency, and use of the latest technology for administering safe anesthesia.^17^

For these reasons, programs that encourage the education of pediatric anesthesia and the work of individuals trained to handle children in resource-limited countries, such as ours, should be supported, as they play an important role in promoting safe anesthesia for children.^15^^,^^16^ Being trained and accredited as a specialist by a recognized institution is the foundation for the future of perioperative care for this vulnerable population.^17^

In Brazil, in the early 1990s, two major pediatric hospitals, Pequeno Príncipe and Joana de Gusmão, were pioneers in offering one-year theoretical and practical training in the subspecialty of pediatric anesthesiology. In recent decades, there has been a growing interest in sub specialization. Currently, we have a few fellowship programs (Pequeno Príncipe, Instituto da Criança-USP, Hospital de Base de São José do Rio Preto, Instituto Nacional da Saúde da Mulher, da Criança e do Adolescente Fernandes Figueira, among others)^16^ and two optional fourth-year residency programs in pediatric anesthesiology recognized by the Ministry of Education (Instituto de Medicina Integral Prof. Fernando Figueira and Hospital Infantil Sabará), an important step in the certification of professionals dedicated to this additional training.

Other important forms of training include workshops, where participants can practice specific skills and competencies for the pediatric population. In 2010, the Pediatric Anesthesia Immersion Course (CIAPED) was created by pediatric anesthesiologists from different regions of the country, with support from the Pernambuco State Society of Anesthesiology (SAEPE), which pioneered the training of practical skills and crisis management with realistic simulation. This course is itinerant, reaching all regions of Brazil and other Latin American countries, with an average of five CIAPEDs held per year and having trained approximately 2500 anesthesiologists to date. More recently, the São Paulo State Society of Anesthesiology (SAESP) created the Advanced Pediatric Anesthesia Support (SAAPed), a workshop offered periodically focusing on crisis situations in pediatric anesthesia through lectures, high-fidelity simulators, and scenario debriefing.

What future measures should be taken to improve education and research in pediatric anesthesia in Brazil?

We believe it is essential to define a minimum curriculum that includes the necessary competencies and skills to safely perform pediatric anesthesia and, similarly, to promote the creation of research networks among specialized professionals. It is undeniable that specialized anesthesiologists can conduct higher-quality research, which directly impacts anesthetic care, increases safety and contributes to better outcomes for this vulnerable population. There is clearly room for improvement, and we owe it to future generations.^18^ Public health policymakers are also expected to consider the needs of children in developing the necessary infrastructure and availability of sufficient research funding.

## Conflicts of interest

The authors declare no conflict of interests. The Curie Artificial Intelligence (https://www.aje.com/br/curie/) was used for English editing purpose.
